# Men’s knowledge and attitudes towards dietary prevention of a prostate cancer diagnosis: a qualitative study

**DOI:** 10.1186/1471-2407-14-812

**Published:** 2014-11-05

**Authors:** Jeremy P Horwood, Kerry NL Avery, Chris Metcalfe, Jenny L Donovan, Freddie C Hamdy, David E Neal, J Athene Lane

**Affiliations:** School of Social and Community Medicine, University of Bristol, Canynge Hall, 39 Whatley Road, Bristol, BS8 2PS UK; Nuffield Department of Surgical Sciences, University of Oxford, John Radcliffe Hospital, Oxford, OX3 9DU UK; Department of Oncology, University of Cambridge, Addenbrooke’s Hospital, Cambridge, CB2 0QQ UK; National Institute for Health Research Bristol Nutrition Biomedical Research Unit, University Hospitals Bristol NHS Foundation Trust, Canynge Hall, 39 Whatley Road, Bristol, BS8 2PS UK

**Keywords:** Diet, Green Tea, Lycopene, Prostatic neoplasms, Qualitative research

## Abstract

**Background:**

Prostate cancer (PC) incidence and progression may be influenced by dietary factors, but little is known about the acceptability of dietary modification to men at increased risk of PC. Qualitative interviews with men participating in the ProDiet study were undertaken to explore the feasibility of implementing dietary interventions for the prevention of prostate cancer.

**Methods:**

An interview study nested within the ProDiet randomised feasibility trial of dietary interventions to prevent a PC diagnosis. Men (n = 133) who previously participated in community based prostate specific antigen (PSA) testing without PC but who were at increased risk of the disease were randomly allocation to both lycopene (lycopene or placebo capsules or lycopene rich diet) and green tea (green tea or placebo capsules or green tea drink) for 6 months. Semi-structured interviews were conducted with participants shortly after randomisation, to investigate attitudes towards dietary modification for PC prevention and dietary information. Interviews were audio-recorded, transcribed and analysed to identify common themes.

**Results:**

Interviews were conducted with 21 participants aged 52-72 years with PSA levels between 2.5 and 2.95 ng/ml, or a negative prostate biopsy result. Most men identified the major causes of cancer in general to include diet, environment, ageing and genetic factors. This contrasted sharply with men’s uncertainty about PC aetiology, and the function of the prostate. Men were confused by conflicting messages in the media about dietary practices to promote health overall, but were positive about the potential of lycopene and green tea in relation to PC prevention, valuing their natural components. Furthermore these men wanted tailored dietary advice for PC prevention from their clinicians, whom they considered a trusted source of information.

**Conclusion:**

Men at elevated risk of PC reported uncertainty about PC aetiology and the role of diet in PC prevention, but enthusiasm for dietary modifications that were perceived as ‘simple’ and ‘natural’. The men looked to clinicians to provide consistent disease specific dietary advice. These factors should be taken into consideration by clinicians discussing elevated PSA results with patients and those planning to embark on future trials investigating dietary modification interventions for the prevention of a PC diagnosis.

## Background

Prostate cancer (PC) is most prevalent among high income countries and diet and lifestyle may play a significant role in its development [1]. A typical ‘Western’ diet (high in meat and fat and low in fruits and vegetables), combined with other lifestyle characteristics (e.g. low exercise levels), is positively correlated with PC incidence and mortality [2, 3]. However, conclusive evidence that dietary modification delays or reduces the risk of developing PC is currently lacking [4] and a randomised trial of vitamin E and selenium was stopped early due to those receiving active treatment not benefiting [5]. Variability in the design (study populations, doses and timings) and quality of research in this area has made interpretation of the findings and the development of PC-specific advice difficult [2]. More high quality research is needed before disease-specific dietary recommendations can be developed [6]. The strongest evidence for PC prevention by diet is for lycopene, a constituent of tomatoes [1]. There have also been a range of observational studies regarding other dietary components (e.g. green tea) which showed reduced prevalence in countries with high consumption [7]. In addition, a number of epidemiological studies have suggested that green tea intake may reduce the risk of prostate cancer [8, 9]. However, current evidence is insufficient to demonstrate the role of green tea consumption in PC prevention [10]. Around a third of men change their diet following a diagnosis of PC to help them manage their disease [11]. Men diagnosed with PC have expressed positive attitudes towards behavioural (diet and exercise) interventions [12] and a need for more information on a “prostate friendly diet” [13].

Interventions to prompt dietary modifications may help reduce subsequent PC incidence men at elevated risk of a PC diagnosis identified through PC screening. Two large population-based trials of PC screening have been published [14, 15] and have provided conflicting evidence about the benefits of PC screening. A recent systematic review [16] has not supported universal screening for PC but prostate specific antigen (PSA) testing continues to be widespread in most European countries [17]. Part of the complexity of PC screening is the poor utility of PSA, as around two thirds of men with an elevated PSA will not have cancer diagnosed on biopsy and lower values of PSA cannot exclude the presence of cancer [18]. These men could potentially benefit from dietary and lifestyle advice to reduce their future risk of PC as there is no standard clinical pathway to manage these individuals. However, the knowledge and attitudes (of men at increased risk of PC) towards dietary modification are unknown as is their willingness to adopt such advice. Better understanding of men’s knowledge of cancer aetiology and their attitudes towards dietary modification for PC could inform health promotion activities for men following testing for this disease as well as inform dietary intervention trials in this area. The aim of this qualitative study is to investigate the knowledge and attitudes of men at increased risk of PC towards the role of diet and this disease and their preferences for relevant information.

## Methods

### Study design

This interview study was nested within the Prostate and Diet (ProDiet) randomised feasibility trial (ISRCTN 95931417). Men (n = 133) who were without evidence of PC but at elevated risk of subsequently developing PC; (PSA levels between 2.5 and 2.95 ng/ml; or above 2.95 ng/ml combined with a negative prostate biopsy result) previously identified as part of community-based PSA testing in the ProtecT (Prostate Cancer testing and Treatment study) [19] were recruited into the ProDiet trial from nine primary care practices in the South West of England, between December 2009 and May 2010. ProDiet was a factorial feasibility trial of a dietary intervention for PC. Trial participants were randomly allocated, in a factorial design, to a six month intervention of both lycopene (lycopene or placebo capsules or 1-2 portions of tomatoes a day) and green tea (green tea or placebo capsules or 3 cups green tea a day). This paper reports results from the interview study that investigated trial participants’ awareness and views of the role of diet in PC prevention. The results of the ProDiet feasibility trial will be reported in subsequent publications.

### Procedure

A sample of 29 ProDiet participants were invited for interview. A purposive sample was selected to ensure that men with a range of characteristics (including a range of trial arms, ages and socio-economic backgrounds) were included. All participants provided their written, informed consent to take part immediately prior to the interview. Interviews were conducted in participants’ homes a mean of 43 days (range 20-61) after randomisation and lasted between 32 and 94 (mean 57) minutes. An interview topic guide was used, informed by the literature, including constructs from the Theory of Planned Behaviour [20] and discussion between study researchers (JH, AL, KA). Participants were asked open ended questions exploring their attitudes towards PC and dietary modifications for disease prevention. Ethical approval was obtained from Trent NHS Multicentre Research Ethics Committee (08/H0405/61).

### Data analysis

Data collection and analysis were conducted in parallel and interviews continued to be conducted until data saturation was reached and no new themes arose from the data [21]. Preliminary findings from early interviews were used to refine the topic guides and explored in later interviews. With written informed consent, interviews were audio-recorded, transcribed in full, and imported into NVivo9 to aid data analysis. Pseudonyms were assigned to participants to maintain anonymity. Thematic analyses [22] identified issues of particular salience for participants and across the dataset, using the constant comparison technique [23]. Transcripts were examined on a line-by-line basis with codes being assigned to segments of the data and an initial coding frame developed. An inductive approach was used to identify participants’ view and beliefs. To enhance analysis and enable team discussion and interpretation, team members (JH and KT) independently coded transcripts; any discrepancies were discussed to achieve a coding consensus and maximise rigour. A consensus about the final list of themes was reached through discussion among the research team (JH, KA and JAL).

## Results

Of the 29 men invited, 21 men (72%) agreed to be interviewed. Participants were all Caucasian and married, with ages ranging from 52-72 years. These men varied in socio-economic status and ProDiet allocation (Table 1). Three major themes were identified during the analyses: knowledge of cancer risk factors; attitudes towards green tea and lycopene for PC prevention; and preferences for dietary information for PC prevention. In the verbatim transcript extracts provided, ‘. . .’ is used to denote a pause and all participants have been assigned a letter as a pseudonym.

**Table 1:** **Demographic details of interview participants** ***(N = 21)***

Characteristic		Frequency
**Age** (years)	50-54	2 (10%)
	55-59	1 (5%)
	60-64	4 (19%)
	65-69	10 (48%)
	70-75	4 (19%)
	mean (SD)	65.4 (3.49)
**Family history of PC**	Yes	2 (9.5%)
	No	17 (81%)
	Don’t know	2 (9.5%)
**Occupational class** ^**a**^	Managerial and professional	7 (33.3%)
	Intermediate	3 (14.3%)
	Working	11 (52.4%)
**Previous prostate biopsy**	Yes	4 (19%)
	No	17 (81%)
**PSA level** (ng/ml)	Mean (SD)	3.0 (1.8)
	Range	2.0 - 9.2
**Randomised ProDiet dietary intervention**	Lycopene Capsules^+^, Green Tea drink	7 (33.3%)
	Lycopene Capsules^+^, Green Tea Capsules^+^	9 (43%)
	Tomato diet, Green Tea Capsules^+^	4 (19%)
	Tomato diet, Green Tea drink	1 (4.8%)

^+^or placebo capsules.

^a^based on participant’s occupation using the three-class system [24].

### Knowledge of cancer risk factors

Cancer in general was believed to have several main causes, including diet, ageing, environmental and genetic factors. Two participants with a family history of cancer stated that cancer arises from ‘internal’ bodily causes with genetic predispositions. Two other men suggested specific environmental causes (for example, pollution or pesticides) or diets containing believed carcinogenic ingredients (for example red meat). Most participants believed that cancer is caused by a combination of internal and external factors including the importance of dietary factors.

“…there can be some genetic things that even obviously be passed down from your family but um overall you get the feeling that it is more to do with your lifestyle and the way that perhaps abuse your body with various things that can bring on some of these cancers, but that’s what the papers say you know I just don’t know” Mr A aged 72.

“It’s something with us in us all that perhaps is prompted by an impact and effect but more than that…Oh I suspect it’s experience, viruses potentially diet as well but what actually prompts may actually vary from person to person” Mr K, aged 64.

The majority of participants believed that lifestyle factors, such as diet, smoking and exercise could impact on the risk of cancer. Only two participants were unsure what individuals could do to prevent cancer developing. Most men expressed beliefs that appropriate self-regulation of an individuals’ health behaviours could, if not reduce the likelihood of developing cancer, strengthen the body’s response to disease.

“For some cancers yes like lung cancer you don’t smoke um but I think uh there are a lot of rumours and myths if you like about certain diets um preventing or helping to prevent certain types of cancer” Mr B, aged 66.

“I imagine keeping you fit I can’t imagine that they would in as such prevent cancer other than they keep you fit and then if you are fit you might then fight off things but other than that, but as a preventative I’m not sure about that” Mr S, aged 69.

In direct contrast, men predominantly expressed a lack of knowledge about the function of the prostate and PC aetiology and commonly expressed anxiety regarding the development of PC, and were consequently unsure about strategies to help prevent PC. These perceptions were similar across all socio-economic groups. The few men who expressed some knowledge about PC most commonly attributed this disease to ageing. Some men expressed understanding of this notion by using ‘body as machine’ metaphors, with ageing contributing to body parts ‘wearing out’ or ‘breaking down’.

“…the last few years we’ve been hearing about prostate cancer more and more like and all we think is oh I don’t want that. But we don’t know what the gland’s there for, we don’t know what it does, so we don’t know what to do” Mr U, aged 70.

“I don’t know, I’ve no idea really. It really does seem you know if there is an organ sort of bug inside us there’s not much we can do about. Um I can’t think of anything that I would do to ward off or help ward off or even you know keep my fingers crossed about prostate cancer or whatever you know” Mr F, aged 66.

“Well I know it’s it’s quite prevalent but the actual cause I don’t know. I always you, one used to associate it with age…but now you know cases are coming to light where men are getting it, appear to you know it’s it’s more publicised that they’re getting it at an earlier age cos you used to think you know you used to think it’d be 60’s and 70’s. Um but now it seems to be 40’s and 50’s you know sort of particularly things like you get publicity about sportsmen and things like that er getting it” Mr D, age 68.

“Growing old as much as anything, I think it’s basically the machinery not functioning as as soundly as it might and therefore one has hope hopefully to to lead a life which copes with that slight run down” Mr K, aged 64.

### Attitudes towards green tea and lycopene for PC prevention

Few men had previously drunk green tea or were aware of any potential health benefits of it before participating in the ProDiet study. Most participants were however, enthusiastic about the benefits of green tea and viewed it as an accessible, ‘natural’, straightforward dietary modification that could easily be adopted into their daily lives. The majority of men did not hold strong preferences for taking green tea either in drink or capsules form; green tea capsules were welcomed for the few men who did not like drinking green tea.

“I was pleasantly surprised that there may be something lurking around on the shelves of the supermarket that could help with the sort of non-event as it were of prostate cancer” Mr A, aged 72.

“I don’t see why not. Very very strong chemicals come from nature and I think we are using them all the time aren’t we, we just don’t realise it? The pharmaceutical companies are supplying them, storing them and testing them by the millions aren’t they from nature just trying to find effective drugs and things, so there is no reason why it shouldn’t come from there” Mr S, aged 69.

“If we heard it was beneficial and it was proved to be so then it’s one of the easiest things in the world to just switch to drinking green tea” Mr M, aged 58.

“I have smelt it. I wouldn’t be. It didn’t really appeal to me. I just thought, you know, I probably wouldn’t. I don’t tea, ordinary tea, anyway. I usually have coffee. So, uh, when they gave you the, uh, little capsules for green tea, that was quite a relief” Mr H, aged 60.

All men were encouraged by the idea that lycopene may provide health benefits in relation to PC prevention. Eating more tomatoes was seen by many to complement existing eating habits and food preferences, with the majority of men reporting that they already regularly ate tomatoes, and so facing little difficulty further increasing their consumption as it could be a simply incorporated into their present lifestyle. A lycopene intervention was regarded as the adoption of a ‘natural’ and ‘fresh’ component to men’ diet, whether in food or capsule form.

“I think it’ll be brilliant when you’ve found out about it if it does, we’ll have to have a tomato and an apple a day” Mr U, aged 70.

“I always ate tomatoes so so that wouldn’t be a problem you know you don’t have to eat supplements, if it’s a proven factor then I would have to consider it” Mr J, aged 72.

“I think anything natural, you know, must must be beneficial. Generally I want it, generally eat natural food rather than processed food whilst we all eat processed food but, um, so yeah I think the more natural you keep it the better. I’m sure there’s some goodness in tomatoes. I mean whether they, help stop cancer I don’t know “It’s quite possible isn’t it. I think anything natural, you know, must must be beneficial” Mr P, aged 68.

### Preferences for dietary information for PC prevention

Participants identified a variety of sources from which they gained health-related dietary information or advice, including the media, the internet and health professionals. Most participants expressed concern and scepticism over the content and conflicting messages in the media about dietary practices to promote their health. A lack of credible scientific evidence to confirm health claims made by the media also made the men wary of acting on such information.

“Oh very often there are conflicting messages come over but when you realise that we get most of our information from the television we don’t look through scientific journals to uh to weigh up the evidence, and if you go on the internet you just get conflicting stories the worst thing you can do is go on the internet, you don’t know which are the reliable sources” Mr M, aged 58.

“I think a lot of reports such as that are made as a result of very limited studies. Um uh where they have found that uh with a small uh a certain sample of the population um drinking blueberry juice or um before that it was cranberry juice um yeah has a beneficial effect or might have a beneficial effect because of antioxidants in those products. Um but you know there’s uh as far as I know there has been no rigorous scientific studies to prove that it’s effective” Mr B, aged 66.

Men typically expressed confidence about knowing what constituted healthy eating, with many believing that they already had a healthy diet and arguing that further general healthy eating advice would, therefore, not be of relevance to them. Most men had, however, already made positive dietary changes in recent years, such as reducing sugar, alcohol and fatty food consumption, often triggered by weight or health issues such as high blood pressure or cholesterol. Dietary changes were typically prompted by, or made in collaboration with, their partner or primary care practitioner.

“Politicians are a bit like newspaper reporters, they’ve got their own agenda…I mean whatever’s again flavour of the month…I tend to ignore them. I’ve always known what’s been healthy for me” Mr L, aged 54.

“I went to the doctor we got to see a nurse up the health centre every six months and have like a MOT, you know blood pressure and that, and she told me I ought to get some weight off. I’d feel better and since then, that’s what I’ve been doing. So I just cut my food down, and since…I’ve cut really right down. I don’t eat big dinners any more, I don’t eat fatty foods, sweets, anything like that now. And I don’t drink much anyway now so, I’ve cut that out a bit as well” Mr I, aged 67.

“I said I want to go on a low fat diet and so my wife agrees because she wouldn’t mind losing a bit of weight as well and so we do we eat the same things more or less” Mr M, aged 58.

“I went to the doctor to ask for a check on my cholesterol um because of my friends who’d died or collapsed or…and um he said that the readings would put me on to drugs as he worded it, I thought he was probably talking about statins. And so I asked if I could try a low fat diet instead to get the reading down and so been on that ever since” Mr M, aged 58.

Most men were open to the idea of making further dietary changes, if the information was targeted to their needs and circumstances and came from a trusted source such as a health professional. The majority of participants stated that they would welcome disease specific dietary modification information for PC and expressed willingness to modify their diet accordingly.

“I don’t want somebody telling me this is what you should be eating when you come home tonight because I think I know that what I eat anyway is quite healthy so I don’t want somebody putting stuff through my letter box…but as I say if it was something that was targeted at me and they thought this was something that was going to help me and they knew me as it were I would be grateful for that” Mr R. Aged 66.

“I think advertising on television and, a- in magazines and what have you has less impact – I wouldn’t say there’s none but there’s less impact but-but if you had a letter from your doctor saying, this survey’s been carried out – this, that and the other - I- I would take, probably, quite a bit of notice of that” Mr O, aged 68.

“I don’t think I would go along with something just because I read it in the paper. If it was if it was information coming from our local GP surgery or one of my visits there then obviously I would go you know I would bear in mind…would believe that source. I wouldn’t necessarily take it from a newspaper” Mr D, age 68.

“You say green tea was going to do me good yeah and then I, say you said it, and then I consulted my doctor and he or she said yes I think there is some there are some good things in that, then I would increase my consumption of green tea and I would have a go… and we get used to things so we would say it’s not too bad now, I’ve got used to drinking it because it’s gonna do me some good” Mr T, aged 65.

## Discussion

Men at increased risk of PC had some understanding of cancer causation, but by comparison limited understanding of PC aetiology, which concurs with previous international surveys [25, 26]. Men recognised the importance of diet in relation to good health and its role in preventing cancers in general. The men interviewed believed that they can make lifestyle changes regarding cancer in general, but were uncertain about what they may be able to do to reduce their risk of developing PC. This study demonstrates that, although men were generally unfamiliar with the potential PC preventative benefits of green tea and lycopene, they were willing to consider using these products (either as tomato rich diet, tea or capsules) as they valued their ‘natural’ qualities and considered them a simple dietary modification that could be easily adopted. Men across all socio-economic groups demonstrated an interest in ‘prostate friendly’ dietary advice and were motivated to implement dietary changes.

The growth in media reports linking diet and PC, has led to increased public interest in disease specific nutritional education and dietary modification [27]. However, the men interviewed were distrustful of dietary information from the media and, similar to previous surveys, overwhelmed by multiple sources of contradictory dietary information [28]. However, most men demonstrated knowledge and interest in healthy eating information, and articulated confidence in the importance of healthy eating for preserving health. The majority of men had made some dietary changes in recent years, usually on the recommendation of their clinician over concerns about their own or their partners’ health. This contrasts with the traditional idea that men are unconcerned with, and reluctant to change, their diet [29] and are dismissive of healthy eating campaign messages [30]. Previous research has suggested that male cancer patients may be reluctant to introduce dietary modification [28, 31], suggesting that gender may be an important determinant of men’s dietary responses to PC. This has been attributed to dietary modifications often being viewed as mimicking “feminine” eating behaviours, such as emphasizing an increase in fruit and vegetables and smaller portion sizes [32]. However, inconsistent media reports regarding the inconclusive evidence around diet, PC and survival rates make it difficult for men to know how to modify their diet. This may contribute to a lack of engagement with dietary modifications [28], as does the requirement to adhere to demanding dietary modifications, such as strict, low-fat, plant-based diets [28].

Our findings suggest that men are sceptical about healthy eating messages in the media, but this is due to their contradictory nature and a lack of trust of the credibility of the source of these messages. However, men did highly value consistent dietary advice and information from a clinician, as this was thought to be more trustworthy, especially if it was personally targeted at them in relation to their lifestyle. It is becoming commonplace for patients to ask clinicians about dietary recommendations for the prostate [2], Clinicians’ can play a valuable role in increasing awareness of the possible benefits of lycopene and green tea, especially as men viewed these as simple, natural and acceptable dietary modifications that complemented established eating habits. Using ‘body as machine’ metaphors could be useful to engage some men in explaining the risk and development of PC and help reduce the anxiety of receiving an elevated PSA level result. This has been used successfully in initiatives such as the ‘*Haynes owner’s workshop manual’* [33] and others that have framed men’s health in terms of mechanical objects, such as cars, requiring tuning [34]. However, a targeted approach to delivering health promotion is requested by men and clinicians should be cautious of using a ‘one size fits all’ approach [35].

This study is one of the first to undertake an in-depth investigation of the views and beliefs of men at increased risk of PC regarding PC aetiology and diet. We used established methods to determine the sample size [21]. Data collection continued in parallel to analysis until data saturation was achieved, that is, no new themes emerging from the data by the end of data collection, the important determinate of sample size in qualitative work. Men from different socioeconomic backgrounds and ages were interviewed enabling a comprehensive insight into men’s views. However, a limitation of the study was that all participants were Caucasian, so future research should include men from non-Caucasian ethnic populations. All participants in this study were married. For some men, their partners had an influence on past dietary changes and may therefore also influence men’s willingness to adopt dietary modifications. Further research to explore this potential ‘partner effect’ and to compare the experiences and perceptions of men with and without partners is required. In addition, participants had previously taken part in the ProtecT study [19], and therefore may have been more informed about PC than men at increased risk for prostate cancer in general. They may also have been more interested in diet as a potential method of preventing a PC diagnosis as they were participating in the ProDiet study. However, results indicated that most men, including those that had received a biopsy, lacked knowledge about PC aetiology and prevention. In addition, men had direct experience of dietary interventions within the ProDiet trial and so were able to give informative views regarding their experiences of dietary modification for PC prevention. Data suggest that dietary modification trials are acceptable to men at elevated risk of PC.

## Conclusions

Men at elevated risk of PC were uncertain about how dietary modification may facilitate prevention of a PC diagnosis and were concerned about conflicting messages about dietary practices to promote their health. Most men would welcome consistent advice from their clinicians about dietary modification to prevent PC. The men reported they were willing to modify their diet if the changes were seen as ‘simple’ and ‘natural’. These factors should encourage clinicians to discuss dietary modifications with patients who have received an elevated PSA level result. The findings may also be taken into consideration by those designing future trials investigating dietary modification interventions for the prevention of PC to help inform clinicians’ dietary messages.
